# Colonic Diaphragm Disease Induced by Chronic Non-steroidal Anti-inflammatory Drug (NSAID)-Use Requiring Multiple Endoscopic Interventions: A Case Report

**DOI:** 10.7759/cureus.85621

**Published:** 2025-06-09

**Authors:** Hasan Jaber, Fawzi Elias, Nader Al Souky, Obada Tayyem, Gebran Khneizer

**Affiliations:** 1 Internal Medicine, University of Kansas School of Medicine, Wichita, USA; 2 Gastroenterology and Hepatology, University of Texas Medical Branch, Galveston, USA; 3 Gastroenterology, Kansas Endoscopy and Gastroenterology, Wichita, USA

**Keywords:** balloon dilation, colonic diaphragm, colonic diseases, colonoscopy, nsaids

## Abstract

Non-steroidal anti-inflammatory drug (NSAID)-induced colonic diaphragm disease is an uncommon yet significant cause of gastrointestinal symptoms. We present a case of a 51-year-old male with chronic NSAID use who developed abdominal pain, weight loss, and gastrointestinal bleeding. Imaging and endoscopy revealed a short, benign-appearing stricture in the ascending colon consistent with diaphragm disease. The patient required multiple endoscopic interventions, including lumen-apposing metal stent placement and balloon dilation, to relieve the obstruction. This case underscores the importance of recognizing NSAID-induced colonic pathology and highlights the role of advanced endoscopic techniques in managing complex strictures.

## Introduction

Non-steroidal anti-inflammatory drugs (NSAIDs) are one of the most commonly used medications, with an estimated 29 million daily users in the United States [1]. While their upper gastrointestinal and intestinal adverse effects are well established, their impact on the colon remains less recognized [2]. NSAID-induced colopathy encompasses a spectrum of manifestations, including colonic inflammation, ulceration, exacerbation of inflammatory bowel disease, complications of diverticulosis, as well as formation of diaphragm-like strictures, termed diaphragm disease [1-3]. Diaphragm disease is characterized by thin, concentric, diaphragm-like strictures within the intestinal lumen, most often resulting from chronic NSAID use. These fibrotic bands can cause variable gastrointestinal symptoms, ranging from subclinical findings to overt obstruction. Despite its rarity, diaphragm disease can lead to severe colonic obstruction, posing diagnostic and therapeutic challenges [2-4].

This article was previously presented as a meeting abstract at the American College of Gastroenterology (ACG) Annual Scientific Meeting on October 27, 2024

## Case presentation

A 51-year-old male presented to the emergency department (ED) with severe lower abdominal pain, infrequent episodes of loose, dark red bloody stools, and an unintentional 40-pound weight loss over three months. He had been taking chronic etodolac.

**Figure 1:**
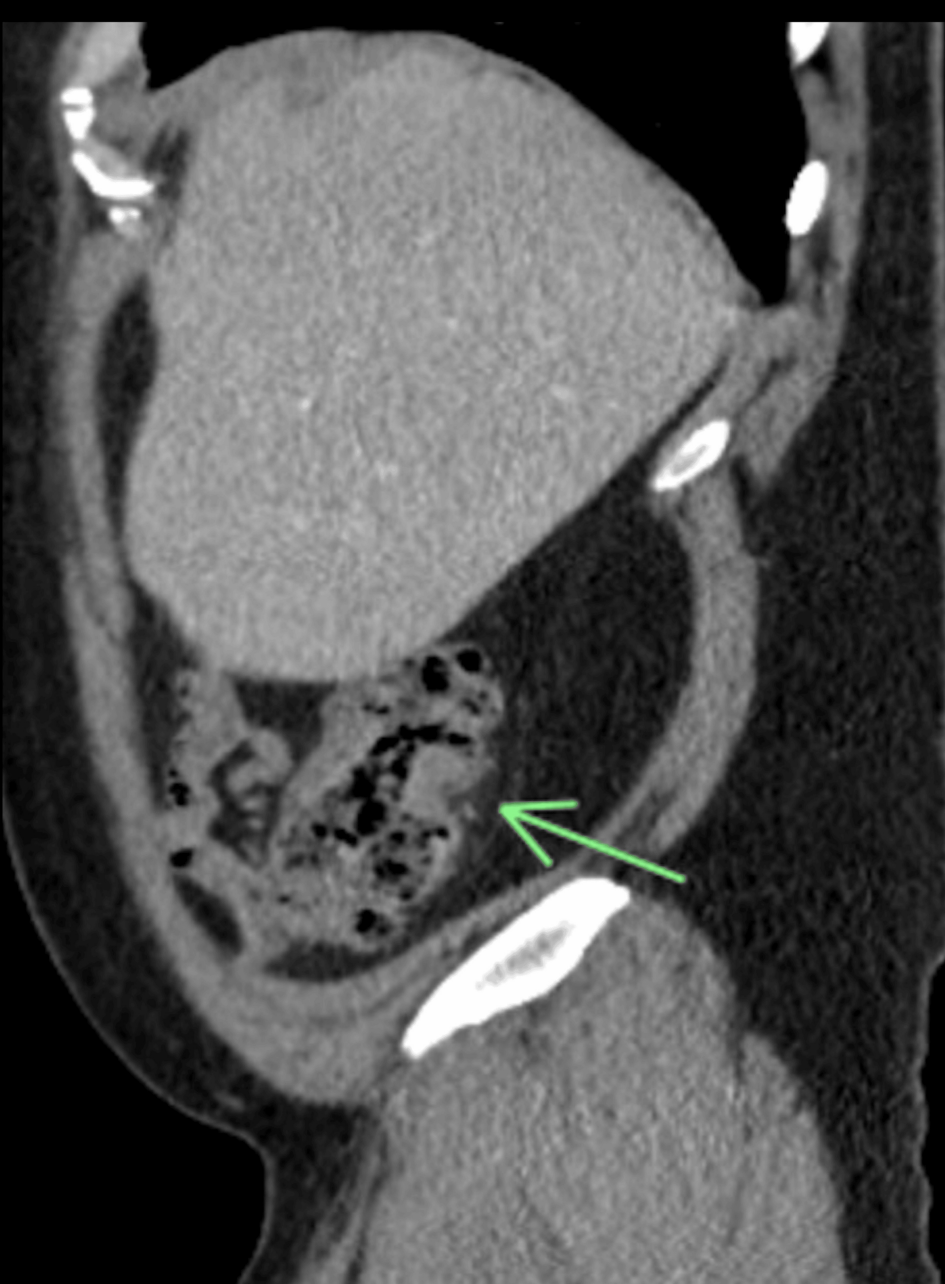
CT scan of the abdomen showing ascending colon stricture Arrow showing localized wall thickening and luminal narrowing of the ascending colon with surrounding inflammation

Laboratory tests revealed a white blood cell (WBC) count of 18,000 cells/µL (4000 - 11,000 cells/µL) and hemoglobin of 8.4 g/dL (13.5 - 17.5 g/dL), while stool studies and celiac serology were unremarkable. A computed tomography (CT) scan of the abdomen showed localized wall thickening and luminal narrowing of the ascending colon with surrounding inflammation (Figure 1).

**Figure 2:**
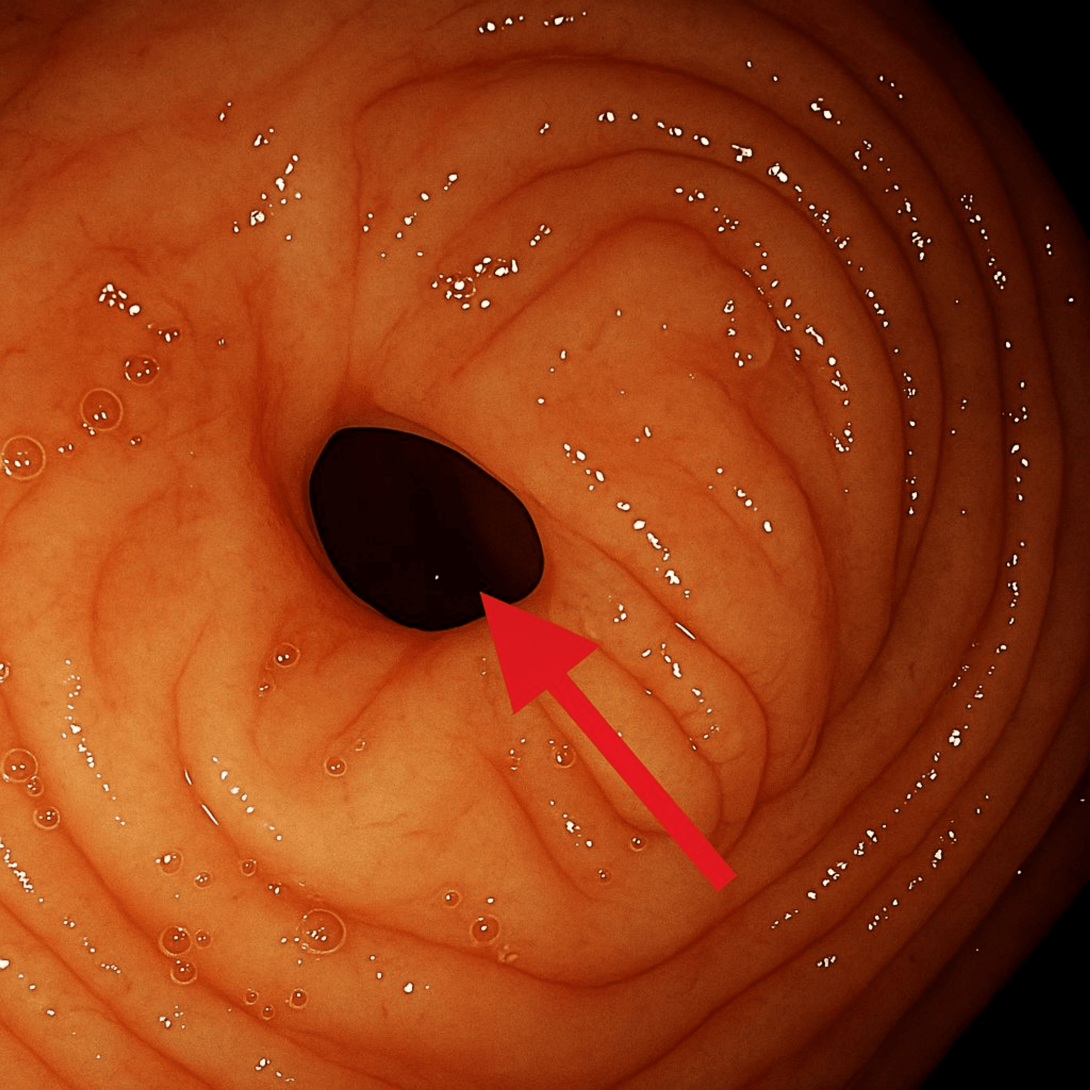
Colonic diaphragm 1 cm in length, 6 mm in diameter before dilatation. Arrow showing the non-steroidal anti-inflammatory drug (NSAID)-induced stricture in the ascending colon.

**Figure 3:**
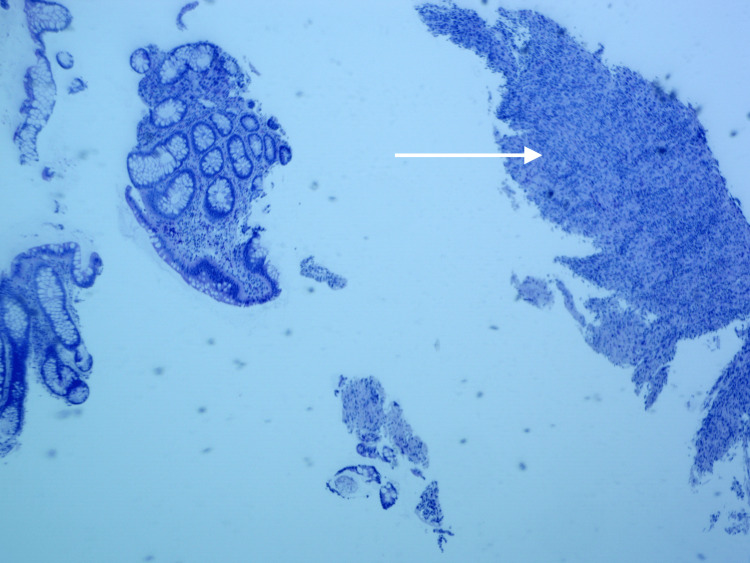
H&E stain under 4x magnification showing crypt drop out within the stricture of the ascending colon Arrow showing crypt drop out

**Figure 4:**
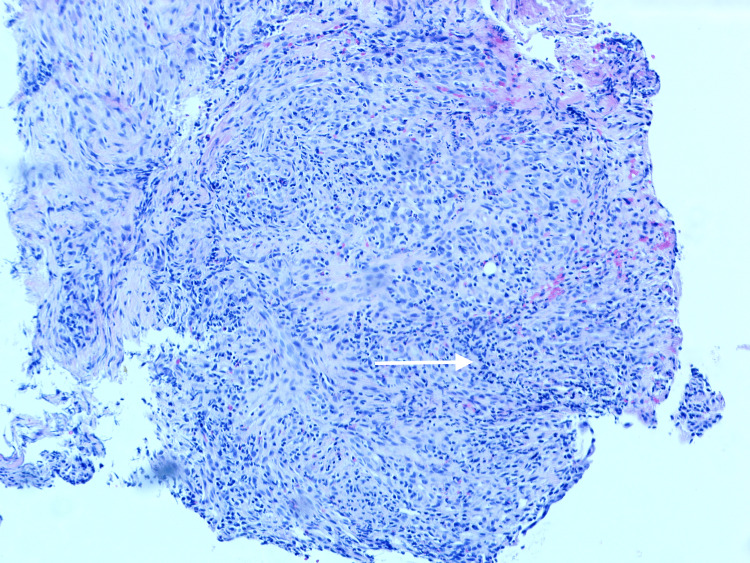
H&E stain under 10x magnification showing focal chronic active colitis in the ascending colon stricture Arrow showing infiltrating inflammatory cells

Colonoscopy revealed a benign-appearing, short stricture (1 cm in length, 6 mm in diameter) in the mid-ascending colon that could not be traversed (Figure 2); therefore, a 20 mm x 10 mm AXIOS™ lumen-apposing metal stent (Boston Scientific, Marlborough, Massachusetts) was placed. Biopsies of the stricture and ascending colon showed crypt drop-out (Figure 3) with focal chronic active colitis but no granulomas or dysplasia (Figure 4). He was then started on prednisone 40 mg daily.

**Figure 5:**
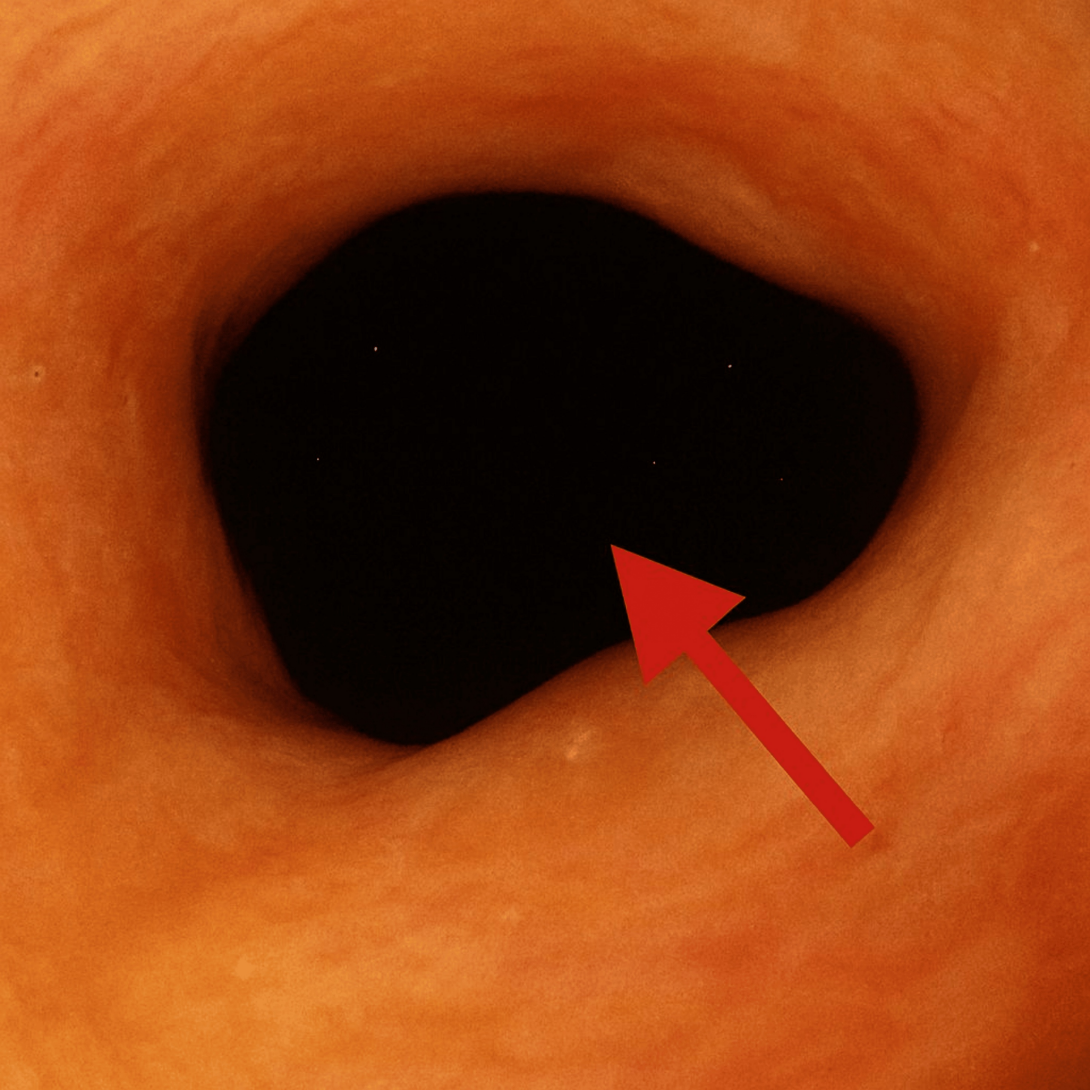
Ascending colon post balloon dilatation Arrow indicating increased colonic diameter following balloon dilatation.

Due to persistent symptoms, a follow-up colonoscopy was performed four weeks later, showing that the stent had migrated. The decision was made to pursue gradual through-the-scope balloon dilation (18-19-20 mm), leading to improved but not complete reversal of luminal narrowing and resolution of his symptoms (Figure 5). The terminal ileum was evaluated after bypassing the stricture, and it was normal.

## Discussion

Diaphragm disease is a rare condition considered pathognomonic of NSAID use [4]. It occurs more frequently in the small intestine, with colonic involvement accounting for only one-third of cases [5]. 

Colonic diaphragm disease most frequently affects the ascending colon, suggesting that mucosal injury may be due to local toxicity from intraluminal NSAID exposure [3-6]. This is supported by the fact that the incidence of NSAID-induced colopathy increased after the introduction of slow-release formulations, which led to increased colonic drug exposure [3]. It is theorized that the high availability of NSAIDs intraluminally causes mucosal injury [5] through different mechanisms, including uncoupling of mitochondrial oxidative phosphorylation and decreased prostaglandin production due to cyclo-oxygenase inhibition [7]. Damage to the mucosal barrier subsequently exposes the mucosa to the noxious intraluminal contents, including bacteria, which would lead to inflammation and subsequent scarring and stricturing [5-7]. Thus, diaphragms are thought to arise from colonic ulceration [3]. Nevertheless, systemic effects of NSAIDs cannot be completely excluded, as demonstrated by a case report describing multiple right-sided strictures in a patient taking rectal indomethacin despite discontinuing oral NSAIDs 10 years prior to symptom onset [8].

Histologically, diaphragms are characterized by submucosal fibrosis with a disorganized (hamartoma-like) arrangement of smooth muscle, blood vessels, nerves, and ganglion cells [4-6]. In some cases, a rim ulcer can be present at the edge of the diaphragm [3], histologically seen as mild acute inflammation [4]. 

The clinical presentation of colonic diaphragm disease is variable and mostly related to either mechanical obstruction or loss of blood and protein from the lesion [3]. Presentation is usually chronic, but instances of acute obstruction and even perforation have been reported [3]. Development of diaphragm disease has been suggested to be more related to the duration of NSAID exposure rather than the medication dosage [9]. However, cases have been reported as early as 18 months after starting NSAIDs [6]. 

Colonoscopy is the primary method of diagnosis, whereby diaphragm-like strictures can be directly visualized in the colon, decreasing false negative results [3-4]. Colonoscopy remains more sensitive than radiologic techniques, as diaphragms may be too thin to be visualized on contrast imaging [3]. 

The first step in managing diaphragm disease typically involves discontinuing NSAIDs [1,5,6], which can alleviate inflammatory symptoms but do not resolve the fibrotic stricture [3]. One exception is a case of four proximal colonic strictures demonstrated endoscopic and histologic resolution three months after NSAID discontinuation [1]. In case the obstruction caused by the stricture needs to be mechanically reversed, the main approaches to therapy are endoscopic dilation or surgical excision [1,3,6]. Notably, severe stenosis may pose challenges to endoscopic intervention, as observed in our case. However, advanced endoscopic techniques, such as through-the-scope balloon dilation or stent placement, may facilitate successful management in such scenarios.

## Conclusions

Colonic diaphragm disease is a rare but significant complication of chronic NSAID use, potentially leading to gastrointestinal bleeding and obstruction. Colonoscopy remains the cornerstone for both diagnosis and treatment. While management ranges from NSAID discontinuation to endoscopic dilation or, in severe cases, surgical resection, some strictures can present considerable challenges. Recognizing this condition early and tailoring the therapeutic approach based on the stricture.
